# Electrode Placement in Transcranial Direct Current Stimulation—How Reliable Is the Determination of C3/C4?

**DOI:** 10.3390/brainsci9030069

**Published:** 2019-03-22

**Authors:** Tonya L. Rich, Bernadette T. Gillick

**Affiliations:** Department of Rehabilitation Medicine, Division of Rehabilitation Science, University of Minnesota, 420 Delaware Street SE, MMC 388, Minneapolis, MN 55455, USA; rich1038@umn.edu

**Keywords:** Electroencephalogram, Non-invasive brain stimulation, stroke, neuroplasticity, transcranial direct current stimulation, adults, children

## Abstract

The 10/20 electroencephalogram (EEG) measurements system often guides electrode placement for transcranial direct current stimulation (tDCS), a form of non-invasive brain stimulation. One targeted region of the brain is the primary motor cortex (M1) for motor recovery after stroke, among other clinical indications. M1 is identified by C3 and C4 of the 10/20 EEG system yet the reliability of 10/20 EEG measurements by novice research raters is unknown. We investigated the reliability of the 10/20 EEG measurements for C3 and C4 in 25 adult participants. Two novice raters were assessed for inter-rater reliability. Both raters received two hours of instruction from a registered neurodiagnostic technician. One of the raters completed the measurements across two testing days for intra-rater reliability. Relative reliability was determined using the intraclass coefficient (ICC) and absolute reliability. We observed a low to fair inter and intra-rater ICC for motor cortex measurements. The absolute reliability was <1.0 cm by different novice raters and on different days. Although a low error was observed, consideration of the integrity of the targeted region of the brain is critical when designing tDCS interventions in clinical populations who may have compromised brain structure, due to a lesion or altered anatomy.

## 1. Introduction

Electroencephalogram (EEG) assessment is a commonly utilized clinical tool for recording brain activity with the use of electrodes placed systematically on the scalp [1]. Clinical EEG assessment has existed for over 50 years relying on EEG technicians and trained medical providers to reliably place recording electrodes to gather clinical data for patient decision making [2,3]. The recent construct of non-invasive brain stimulation trials integrates the 10/20 International Electroencephalogram Coordinate System (10/20 EEG) as a means to guide electrode placement for transcranial direct current stimulation (tDCS) over the motor cortex (generally the C3/C4 region).

TDCS is a novel approach to rehabilitation under investigation in both children and adults. TDCS influences the excitability of the brain with use of electrical stimulation via sponge electrodes placed on the scalp in different electrode montages to target various regions of the brain (i.e., motor cortex, dorsolateral prefrontal cortex, etc.) [4]. Standard tDCS involves the placement of two electrodes, including an anode and a cathode at a location guided by the 10/20 EEG system. TDCS electrode montages include primary motor cortex (M1)-supraorbital fossa (SO); bihemispheric with electrode placement on the bilateral M1 (C3/C4) or bilateral dorsolateral prefrontal cortex (F3/F4) [4]. Investigators select regions of the brain hypothesized to contribute to a targeted function (e.g., motor, cognition, pain control) [4]. The results of tDCS clinical trials suggest a strong safety profile (i.e., no serious adverse events reported in the literature) and a demonstrated ability to provide stimulation simultaneously with rehabilitation tasks [5,6].

Recent translational neuroscience studies investigate brain activity as relates to developing cognition over the lifespan and measuring recovery from brain injury with the use of EEG electrode caps [7,8]. EEG electrode caps can be placed quickly. Some commercially available EEG systems can be placed on the participant without head-specific measurements with recent portable EEG systems showing validation and cost-effectiveness over traditional EEG units [9]. Studies are now using a combination of EEG cap recording prior to or following non-invasive brain stimulation to differentiate responders to neuromodulation interventions [10,11]. Given the ease of commercially available EEG cap systems, investigator training requirements can be minimized.

TDCS is a neuromodulatory intervention that is increasingly incorporated into the investigation for cognitive skills in neuropsychiatric conditions [12], pain control for neuropathic pain [13], motor recovery after stroke [6], among other clinical conditions. Specific to stroke, a common electrode placement targets the M1 for the recovery of motor function after stroke. Two methods exist for somatotopic localization of the M1 area for tDCS electrode placement, non-invasive brain stimulation testing using transcranial magnetic stimulation (TMS) and the 10/20 International Electroencephalogram Coordinate System (10/20 EEG). TMS is useful when the underlying anatomy may be altered due to pathology, such as in individuals with stroke. Meaning, the M1 may have reorganized and be located in an alternate location, due to a stroke lesion therefore relying on scalp-based measurements may have poor validity [14]. TMS requires additional equipment for testing (i.e., electromagnetic coil, stereotactic neuronavigation that incorporates individual magnetic resonance imaging-MRI). For clinical populations without anticipated altered anatomy, the 10/20 EEG system may be a more cost-effective means for localizing the desired coordinates (such as M1) for tDCS electrode placement.

The coordinates for M1 include C3 (left hemisphere) and C4 (right hemisphere) regions [15]. To apply the 10/20 EEG methodology for identifying C3 and C4, the 10/20 EEG method identifies C3 and C4 from four key individual anatomical landmarks including the nasion (lowest depression between the forehead and nose), inion (lowest point of the skull from the back of the head) and the preauricular points of each ear. Using these anatomical landmarks, it is possible to derive the coordinates that approximate different regions of the brain [1].

Although the 10/20 EEG electrode system was originally designed to record brain activity, published investigations of tDCS in both adults and children are reporting the use of the 10/20 EEG system to guide tDCS electrode placement [12,16]. However, the reliability of the 10/20 EEG measurements by novice investigators to locate the C3 and C4, as a marker of the relative underlying location of the primary motor cortex (M1), is unknown. The purpose of this study was to assess the inter-rater and intra-rater reliability of the 10/20 EEG system to localize C3 and C4 to provide information about the error of this measure when used by a researcher without formal training in EEG measurements. Our intention was to inform the study design of NIBS research protocols and electrode placement training.

## 2. Materials and Methods

This research was approved by the Institutional Review Board at the University of Minnesota. A convenience sample of twenty-five adult participants without neurological conditions was recruited (mean age = 42.04 years, SD = 13.67 years). No prior studies comparing the reliability of 10/20 EEG measurements exist. Therefore, the study sample size is based on practical considerations including participant flow and the number of participants needed to reasonably evaluate reliability. All participants provided written consent.

In preparation for this study, a registered neurodiagnostic technician (R. EEG. T) trained two investigators unfamiliar with performing head-specific 10/20 EEG measurements. Each investigator completed approximately two hours of training with the technician. Training was comprised of instruction on C3 and C4 localization using standard measurements with a tape measure and return demonstration by the two investigators for the registered neurodiagnostic technician.

**Figure 1:**
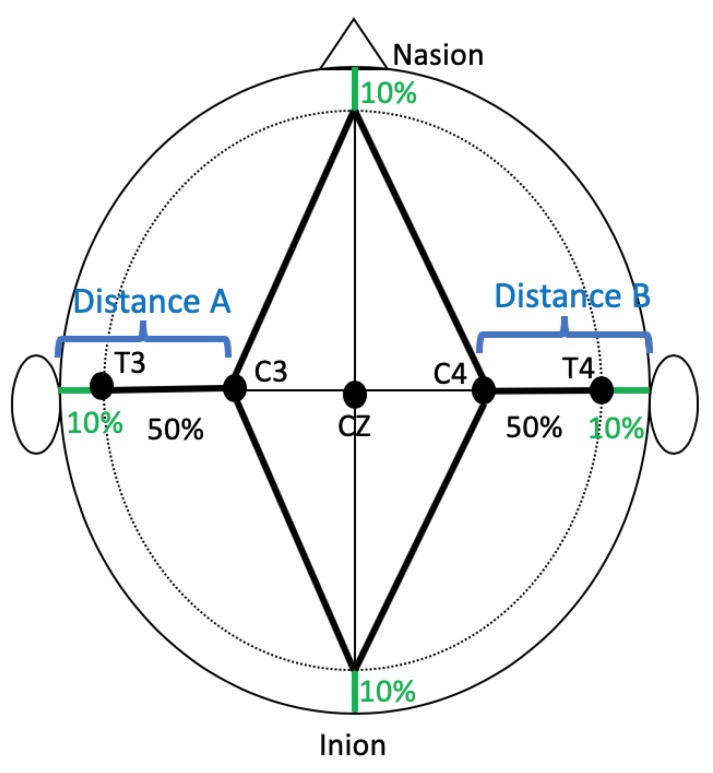
Motor cortex localization using the 10/20 Electroencephalogram (EEG) System. Traditional, manual 10/20 EEG measurements to localize the motor cortex of the left hemisphere (C3) and right hemisphere (C4) based on structural landmarks of the head. Landmarks are marked including the nasion, inion, and preauricular notches on the head to guide measurements. Percentages represent the percentage of the distance from the landmark to the EEG location. The measurement of interest is Distance A, from the preauricular notch to C3, and Distance B, from the preauricular notch to C4. 10/20 EEG: 10/20 International Electroencephalogram Coordinate System, C: Central, CZ: Central Zero; T: Temporal.

For this study, the investigators performed independent, head-specific measurements of participant’s heads following the 10/20 EEG guidelines and training previously provided [17]. The C3 and C4 areas were localized and the distances between (1) left preauricular notch to C3 and (2) right preauricular notch to C4 was measured consistent with clinical practices for localizing C3 and C4 (Figure 1) [1]. Multiple measurements (i.e., head circumference, nasion to inion, etc.) are taken to derive the location of C3 and C4. The measurements of interest for this study were the distance from the preauricular notch to C3 (Distance A on Figure 1) and C4 respectively (Distance B on Figure 1).

For inter-rater reliability, two raters measured all 25 participants on the same day. For intra-rater reliability, only one rater evaluated the same 25 participants on two consecutive days. The relative reliability was determined using the Intraclass Correlation Coefficients (ICCs) and the absolute reliability was determined by calculating the standard error of measurement (SEM) (SEM = $\sqrt{WMS}$, where the within-subject mean (WMS) is the square error term from 1-way analysis of variance). The ICC values were considered poor when below 0.20, fair from 0.21 to 0.40, moderate from 0.41 to 0.60, good from 0.61 to 0.80, and very good from 0.81 to 1.00 [18].

## 3. Results

The ICC analyses were performed using SPSS Version 17 (SPSS Inc, Chicago, IL, USA) and the SEM was calculated using Excel 2007. The ICC for the inter-rater reliability of the distance between left preauricular notch to C3 was 0.36 (95% confidence interval = −0.03–0.65) and the SEM was 0.50 cm. The ICC for the inter-rater reliability of the distance between right preauricular notch to C4 was 0.05 (95% confidence interval = −0.34–0.43) and the SEM was 0.58 cm. The ICC for the intra-rater reliability of the distance between left preauricular notch to C3 was 0.36 (95% confidence interval: −0.4–0.66) and the SEM was 0.34 cm. The ICC for the intra-rater reliability of the distance between right preauricular notch to C4 was 0.60 (95% confidence interval: −0.27–0.80) and the SEM was 0.34 cm.

## 4. Discussion

This study evaluated the inter and intra-rater reliability of C3 and C4 locations using the 10/20 EEG system by researchers who are a novice to EEG electrode measurements. The study showed a low to fair inter- and intra-rater ICC for the distances between left preauricular notch to C3 and between right preauricular notch to C4. The ICC depends in part on the between-subject variability of the measurements, therefore the low to fair inter- and intra-rater ICC could be attributed to low variability in the head size of these adult study participants. In our study, the SEM was less than 1 cm. The low SEM indicates a small amount of absolute error of the measurement within and between raters. Our results suggest that the use of the 10/20 EEG may result in a low SEM when localizing C3 and C4 by different novice raters and in different days.

Procedurally, the tension of the tape measure and hair management during all measurements was not standardized. Tape measure tension could have been impacted when measuring participants with thick hair. Additionally, a non-toxic marking pen (China™ pencil), which was used for this study to mark head-specific measurements. This non-toxic marking pen was selected to emulate clinical practices. Although clinically relevant, the non-toxic marking pen has a broad tip. Together, these potential sources of variability could have contributed to the errors in the measure. Despite these limitations, our study indicates that after training by a registered neurodiagnostic technician the 10/20 EEG can result in low SEM, when localizing C3 and C4, by different novice raters and on different days. Although limitations exist within this study design, this study advances our understanding of the reliability of the 10/20 EEG when used by multiple NIBS investigators and on different days.

The 10/20 EEG frequently guides electrode placement for tDCS although no consensus on training or reliability of 10/20 EEG measurements for research has been established. A recent study compared the location of the dorsolateral prefrontal 10/20 EEG coordinates using multiple methods [19]. The authors compared the manual measurements of five raters on six participants [19]. The results suggest that although a small difference in location was found, manual measurement differences between raters were not significant and two weeks of training was sufficient to reliably localize the dorsolateral prefrontal cortex. Our results also support the reliability of the 10/20 EEG system following fairly limited instruction. In our study, both investigators were previously rehabilitation clinicians therefore their familiarity with general measurement systems might be higher than a rater who is unfamiliar with measurement procedures.

The study was limited by a lack of three-dimensional analysis of disparities in our inter-rater and intra-rater measurements. Future studies could consider a three-dimensional analysis that could provide an indication of the direction of the displacement error (e.g., anterior, superior, posterior) of C3 and C4 locations observed between inter and intra-raters. Our study focused on adult participants and the reliability of 10/20 EEG measurements in young children experiencing growth in their skull size would be of interest considering the possibility of applying the 10/20 EEG system for tDCS electrode placement in young children.

The reliability and training experience of investigators in the 10/20 EEG measurements is rarely reported in clinical trials. Poor reliability of investigators could suggest inaccurate localization of the targeted region for intervention. Including the amount of 10/20 EEG training and general content of the training in clinical trials would advance the research practice for all investigators.

A recent modeling study determined that electrode placement would be optimally placed within <1.0 cm across sessions to achieve the desired electric field distribution [20]. Our study results suggest that <1.0 cm of precision can be achieved to localize C3 and C4 with novice raters. However, a broader understanding of the clinical significance of precise tDCS electrode placement of a targeted area is not fully understood. Multiple factors impact the electric field including changes in posture, age-related changes in anatomy, and developmental changes in underlying neural tissue [16,17,21,22]. Future studies that incorporate modeling can aid in discerning the effect of precision on electrode placement in clinical trials.

As we consider not only reliability, but also validity, other studies suggest poor validity of the 10/20 EEG measurements to localize the motor cortex in adults without neurological condition [23,24]. The 10/20 EEG system is currently used to guide tDCS electrode placement in studies that include clinical populations without underlying structural integrity (e.g., M1), such as individuals with stroke [25,26]. The influence of development in the motor cortex in diagnoses, such as stroke could limit the 10/20 EEG to accurately locate the M1 area. In clinical populations, reliability of electrode placement is integral. Following confirmation of the M1 using TMS in clinical populations, such as stroke, marking the corners of the electrodes with a non-toxic marking pen between sessions could increase the reliability of electrode placement between sessions as recommended by others [4,27]. Future studies incorporating neuroimaging, stereotactic neuronavigation and TMS as a cortical excitability assessment tool may elucidate the validity of the use of 10/20 EEG to locate the M1 in clinical populations with altered underlying anatomy. Furthermore, investigating the influence of neuromodulation by comparing two methods of somatotopic localization of M1 (TMS vs. 10/20 EEG) in future clinical trials could further elucidate any differences between the two methods.

## 5. Conclusions

Our study investigated the reliability of the 10/20 EEG system by novice research raters in a healthy adult population with stable cranium sizes. In our study, the results suggest a low to fair inter- and intra-rater ICC for the somatotopic localization between preauricular notch to C3 and C4. We observed an SEM of less than 1 cm by different novice raters on different days. These results suggest that novice raters can be provided with training (i.e., 2 h) in the 10/20 EEG system to localize the motor cortex. Reporting the 10/20 EEG measurement reliability and investigator training in clinical trials could systematize 10/20 EEG derived tDCS electrode placement methodologies between studies. Understanding these potential sources of variability could advance the methodology for populations with intact underlying anatomy.
